# Migrant nurses in Brazil: demographic characteristics, migration flow and relationship with the training process

**DOI:** 10.1590/1518-8345.0390.2686

**Published:** 2016-03-28

**Authors:** Kênia Lara Silva, Roseni Rosângela de Sena, Tatiana Silva Tavares, Stephanie Marques Moura Franco Belga, Lucas Wan Der Maas

**Affiliations:** 1 PhD, Professor, Escola de Enfermagem, Universidade Federal de Minas Gerais, Belo Horizonte, MG, Brazil; 2 Doctoral student, Escola de Enfermagem, Universidade Federal de Minas Gerais, Belo Horizonte, MG, Brazil. Scholarship holder from Conselho Nacional de Desenvolvimento Científico e Tecnológico (CNPq), Brazil; 3 Undergraduate student in Health Services Management, Escola de Enfermagem, Universidade Federal de Minas Gerais, Belo Horizonte, MG, Brazil; 4 Doctoral student, Pontifícia Universidade Católica de Minas Gerais, Belo Horizonte, MG, Brazil. Scholarship holder from Coordenação de Aperfeiçoamento de Pessoal de Nível Superior (CAPES), Brazil

**Keywords:** Migration, Nursing Staff, Education, Nursing

## Abstract

**Objective:**

to analyze the migration of nurses in Brazil, describe the demographic characteristics of migrant nurses, the main migration flows, and establish relationships with the training process.

**Method:**

a descriptive, exploratory study, based on 2010 Census data. The data were analyzed using descriptive statistics.

**Result:**

there were 355,383 nurses in Brazil in 2010. Of these, 36,479 (10.3%) reported having moved compared to the year 2005: 18,073 (5.1%) for intrastate migration, 17,525 (4.8%) interstate migration, and 871 (0.2%) international migration. Females (86.3%), Caucasians (65.2%), and unmarried (48.3%) nurses prevailed in the population, without considerable variation between groups according to migration situation. The findings indicate that the migration flows are driven by the training process for states that concentrate a greater number of courses and positions in undergraduate and graduate studies, and the motivation of employment opportunity in regions of economic expansion in the country.

**Conclusion:**

it is necessary to deepen the discussion on the movement of nurses in Brazil, their motivations, and international migration.

## Introduction

In Brazil, the nursing workforce is made up of professional nurses, nurse technicians and nurse auxiliaries. According to the Federal Council of Nursing, 1,535,568 nursing professionals were registered in March of 2011: 314,127 (20.46%) professional nurses, 698,697 (45.5%) nurse technicians, and 508,182 (33.09%) nurse auxiliaries. Of this registered workforce, 55.67% were in the southeast, 19.61% in the northeast, 15.82% in the south, 5.45% in the north, and 3.45% in the Midwestern portion of the country^(^ 1 ^)^.

In the last two decades there has been a movement to expand nursing education in Brazil, with the creation of schools in all regions. Data on enrollments in undergraduate nursing programs show that there has been an increase of approximately 100% in four years, considering data from 2004 to 2008, when enrollment rose from 120,851 to 224,330^(^ 2 ^)^. This movement results from the democratization of access to higher education, and results in more availability of nurses in the marketplace.

Despite the size of the nursing workforce, in absolute numbers, and the increase of the number of nursing schools in the last decade in the country, the concentration of schools and the availability of nurses per capita are quite uneven in different regions^(^ 3 ^)^. Nursing schools are concentrated in the states that are most densely populated and which have the greatest income concentration, accompanying the distribution of gross domestic product (GDP) and economic and social inequalities of the Brazilian regions.

The health labor market has grown in the past two decades in both the public and the private sectors. Among the factors that contributed to this growth are the implementation of the Unified Health System and the change in the care model, with the creation of new jobs, especially in primary care^(^ 4 ^)^. In this sense, the Family Health Strategy (FHS) has represented an important expansion of the health labor market.

The growth of FHS in Brazil is accompanied by opportunities and challenges for the training of health and nursing professionals, both regarding the quantitative aspect, the distribution of professionals, and the possibilities of qualification of the professionals in the labor market^(^ 3 ^)^.

The increase in jobs, however, has not accompanied the expansion of nursing schools and vacancies in undergraduate courses to the same extent in Brazil. These aspects raise questions about the relationship between the expansion of nursing schools, market growth and migration of nurses in Brazil. Due to the increased number of programs, Brazil is referred to as a country with a large supply of these professionals. It is therefore necessary to monitor the labor market and the flow of nurses within and out of the country^(^ 5 ^)^.

Elsewhere in the world, the nursing labor market has often been characterized by numerous imbalances, especially in relation to unemployment and underemployment. Imbalances are characterized by governmental actions that usually influence the supply of health professionals in response to political issues, driven by financial concerns, rather than the rationality of the market or epidemiological reality^(^ 6 ^)^. In Brazil, the labor market for health professionals in general, which includes nurses, has been facing a crisis characterized by the deficit of professionals and the regional, national and sub-national inequality in the distribution and access to the health workforce, particularly affecting the rural areas, urban neighborhoods, or those that are difficult to access^(^ 7 ^)^.

Studies have shown that factors linked to job prospects, such as better working conditions and quality of life, are some of the key issues that influence the migration of health professionals^(^ 5 ^-^ 6 ^,^ 8 ^-^ 9 ^)^. The geographical distribution of these migration movements shows a concentration of professionals in the capital and areas of economic development^(^ 10 ^)^. This process must be analyzed considering its complexity, since high levels of migration can cause problems such as rising unemployment, conflicts between migrants and natives, in addition to the reduction of the workforce in the areas of emigration.

The objective of this study was to analyze the migration of nurses in Brazil, by describing the demographic characteristics of migrant nurses, the main migration flows, and establishing relationships with the training process

## Method

This was a descriptive, exploratory study. Data were obtained from the Census of the Brazilian Institute of Geography and Statistics (IBGE) in 2010. The Census is conducted with the entire population of the country, which is asked basic demographic questions. For a sample of about 10% of the population in the census, broader questions are asked on migration conditions, education, labor, income, and fertility^(^ 11 ^)^.

In this study, data from the Census sample were used, which stand out a priori for the possibility of identifying the population of nurses through the education and work criteria. Nurses were identified in the Census data based on two criteria: (i) degree in nursing, according to the latest college degree completed (ii) occupation, with the selection for the main role of the reference week being "higher level nursing professional". Thus, not only nurses working in the profession are included, but also nurses employed in other roles, along with those who are unemployed, and those who are not economically active.

Based on the defined criteria, cases corresponding to around 470,000 nurses, living in Brazil in 2010, were identified. Although this number does include those that are not economically active, it proved far superior to the records of the Federal Nursing Council (COFEN) at the time of census completion, which was about 300,000. In this sense, consistency was applied to the data, disregarding the cases with education that was lower than an undergraduate level degree, and those aged less than 21 years. This exercise is particularly necessary in the case of nursing, due to the high number of nurse technicians and assistants who registered with the occupation of "higher degree nursing professional", although the Census also offers the option "mid-level nursing professional" in its occupational classification.

After reviewing for consistency, a sample of 30,655 cases identified as nurses was obtained. By expanding the sample, this number would amount to little more than 350,000 nurses in the total population, a contingent similar to the data of 314,127 nurses presented by COFEN at the time^(^ 1 ^)^, considering that nurses who are not economically active or are not serving in the profession are also included in the census. For this reason, data from the Census sample were considered valid for conducting analyses of the higher level nursing workforce and, consequently, of their migration profile. In addition, statistical tests were performed (t-test, chi-square and analysis of variance - ANOVA), which returned significant results for all sample estimators used.

The migration status of nurses was defined from the comparison between the municipalities of current residence, namely the one corresponding to the reference week of the 2010 Census, and the city or country of residence in 2005. Those residing in the country in 2010 and in another country in 2005 were considered as*international migrants*; those who lived in different municipalities in both periods were considered as *interstate migrants*, given that those municipalities were situated in different states; and those who lived in different municipalities of the same Federation Unit (FU) or State in the comparison of both periods were considered *intrastate migrants*. Nurses were also classified as *non-migrants*, i.e., those who have always lived in the current city and *non-migrants in the considered period*, which are those living or having lived in a municipality other than the birth one, but who migrated before 2005.

The variables analyzed relating to the general characteristics of the population of nurses were: (i) demographic: gender, age, race/skin color, and marital status; (ii) education: highest completed course, and course attending at the time of the Census (undergraduate, specialization, master's, doctorate, or, did not attend); (iii) work and income: activity status, mean income for all jobs, and mean household income; and, (iv) migration: migration status, according to previous definition.

The migration flows were calculated by comparing the state of origin (in 2005) and destination (in 2010). Therefore, the number of immigrants, emigrants and the balance are presented. In addition, the calculation of Net Migration Rate (NMR) was calculated by state, given by the ratio between the net migration in the period and the population of nurses in the last year. Positive values ​​indicate that the FU received more migrants than people who emigrated, and negative values ​​indicate the opposite.

## Results

The Census found that there were 355,383 nurses living in Brazil in 2010, an amount corresponding to the expansion of the sample of 30,655. Table 1 shows the general characteristics of the population of nurses, according to migration status.

**Table 1:** - General characteristics of the population of nurses, according to migration status. Brazil, 2010

**Characteristic**	**Non-migrant**	**Non-migrant in the selected period**	**Intra-state migrant**	**Inter-state migrant**	**International migrant**	**Total**	
Sample (n)	13.843	13.126	1.961	1.652	73	30.655	
Expanded sample (N)	165.698	153.216	18.073	17.525	871	355.383	
Female gender (%)	86.1	86.9	84.0	85.2	80.8	86.3	
Mean age, in years	34.5	39.9	31.8	32.4	42.6	35.6	
Race/skin color (%)							
	White	64.1	65.9	67.5	66.7	66.9	65,2
	Black	6.8	5.4	4.4	4.7	2.4	6,0
	Asian	1.3	1.7	1.2	1.1	10.3	1,5
	Mixed race	27.7	26.9	26.7	27.4	20.3	27,2
	Indigenous	0.1	0.2	0.2	0.2	0.0	0,1
Marital status (%)							
	Married	36.8	43.1	40.5	40.8	56.3	39,9
	Divorced/separate	8.3	10.0	7.2	7.1	9.6	8,9
	Widow(er)	2.2	3.7	1.5	1.9	0.5	2,8
	Single	52.8	43.1	50.8	50.2	33.7	48,3
Course attending (%)							
	Higher level	6.5	4.7	3.8	4.5	11.0	5,5
	Specialist	9.4	8.8	11.1	12.8	7.1	9,4
	Master's	1.3	1.1	1.2	1.3	0.9	1,2
	Doctorate	0.3	0.3	0.2	0.5	0.0	0,3
	Does not attend school	82.5	85.1	83.7	80.9	80.9	83,6
Mean time living in the municipality, in years	-	18.8	1.9	2.1	1.4	14.2	
Activity status (%)							
	Employed in the profession	47.3	45.6	50.9	46.5	17.8	46.7
	Employed in another function	34.7	35.5	33.9	29.9	33.3	34.8
	Non-economically active	12.0	14.5	10.2	14.6	42.2	13.2
	Unemployed	5.9	4.4	5.0	8.9	6.8	5.3
Mean income in all jobs (R$)	2.346.23	2.819.39	2.515.33	2.650.17	2.107.52	2.341.17	
Mean household income (R$)	6.092.62	6.711.29	6.143.91	6.209.42	4.782.12	5.798.41	

Source: the authors based on the IBGE Census

Of the total number of nurses living in the country, 36,479 (10.3%) reported movement, compared to 2005: with 18,073 (5.1%) for intrastate migration, 17,525 (4.8%) for interstate migration, and 871 (0.2%) for international migration.

Females (86.3%), Caucasians (65.2%), and unmarried (48.3%) nurses prevailed in the population. There were no considerable variations among groups according to the migration status with regard to these characteristics. However, the higher proportion of blacks and single individuals among nonmigrants, and Asians and married among international migrants, is worth noting. The mean age was 35.6 years, with greater variation in groups of intrastate migrant nurses (31.8 years) and international migrants (42.6 years).

The data show that at the time of data collection, 83.6% of the nurses did not attend school; 9.4% were enrolled in specialist courses, 5.5% in other undergraduate courses, and 1.5% in masters or doctoral courses. The proportion of those enrolled in specialist courses proved greater among intrastate migrants (11.1%) and interstate migrants (12.8%). The proportion of those attending other undergraduate courses was higher among non-migrants (6.5%) and international migrants (11%).

Regarding occupancy status, 46.7% of nurses had jobs in the profession, with the highest rate among intrastate migrants (50.9%) and the lowest among international migrants (17.8%). It was highlighted that 34.8% of nurses had jobs in another role, which did not vary according to immigration status. The overall proportion of unemployed was 5.6%, also with little variation, except among interstate migrants (8.9%) and international migrants (6.8%), which also showed no economically active ratios above the general.

The mean income of nurses in all jobs was R$ 2,341.17 and the mean household income was R$ 5,798.41. The analysis of income according to immigration status did not have significant differences, except for international migrants, who had lower mean incomes.

Table 2 shows the data of the population of immigrants and emigrant nurses, net migration and liquid net migration rate among the states and regions of the country.

**Table 2:** - Population of nurses, immigrants, emigrants, net migration and liquid net migration rate per state and region. Brazil, 2010

	**Population**	**Immigrants**	**Emmigrants**	**Migration net**	**Liquid net of migration**
Rondônia	1,711	135	80	55	3.2
Acre	1,119	144	12	133	11.9
Amazonas	5,119	372	250	122	2.4
Roraima	553	203	27	176	31.9
Pará	6,296	550	407	143	2.3
Amapá	860	88	44	44	5.1
Tocantins	3,236	433	299	134	4.1
North	18,894	1,925	1,119	807	4.3
Maranhão	8,526	510	346	164	1.9
Piauí	4,565	205	436	-231	-5.1
Ceará	11,050	636	471	165	1.5
Rio G. Norte	4,227	305	70	234	5.5
Paraíba	6,661	329	847	-517	-7.8
Pernambuco	10,655	591	670	-80	-0.7
Alagoas	3,006	68	132	-64	-2.1
Sergipe	2,521	287	92	195	7.7
Bahia	21,059	1,394	758	636	3.0
Northeast	72,271	4,324	3,822	502	0.7
Minas Gerais	40,944	1,336	2,583	-1,247	-3.0
Espírito Santo	6,924	320	360	-40	-0.6
Rio de Janeiro	41,077	1,044	1,390	-346	-0.8
São Paulo	100,307	2,676	3,174	-498	-0.5
Southeast	189,251	5,375	7,507	-2,131	-1.1
Paraná	19,090	1,050	1,118	-68	-0.4
Santa Catarina	9,972	967	569	397	4.0
Rio G. Sul	19,979	521	965	-444	-2.2
South	49,041	2,538	2,652	-115	-0.2
Mato G. Sul	4,027	540	327	213	5.3
Mato Grosso	4,498	685	240	445	9.9
Goiás	10,876	712	625	87	0.8
Distrito Federal	6,524	714	522	192	2.9
Midwest	25,926	2,652	1,714	938	3.6

Source: the authors based on the IBGE Census

In absolute numbers, São Paulo (3174) is the state with the largest number of migrants, followed by Minas Gerais (2583), Rio de Janeiro (1390), Paraná (1118) and Rio Grande do Sul (965). The state with the largest number of immigrants is also São Paulo (2676), followed by Bahia (1394), Minas Gerais (1336), Paraná (1050) and Rio de Janeiro (1044). When analyzing the net rate of migration, it is observed that the states of Roraima (31.9), Acre (11.9), Mato Grosso (9.9), Sergipe (7.7) and Rio Grande do Norte (5 5) exhibited the highest positive results. In contrast, the states of Paraíba (-7.8), Piauí (-5.1), Minas Gerais (-3.0), Rio Grande do Sul (-2.2) and Alagoas (-2.1) showed the highest negative rates.

Migratory behavior is predominantly observed, especially for flows from the southeastern to the northeastern and midwestern areas of the country; as well as flows from the northeast to the southeast. Please note that the southeast is the region with the highest number of individuals who leave their place of residence to settle in another region (-2131), evidenced by the negative net migration, followed by the south (-115). Regions that receive more nurses, revealed by positive net migration, are the midwest (938), north (807) and northeast (502).

International migration was reported by 0.24% of the sample (73 cases). Considering the expanded sample, these cases accounted for 871 nurses. This result reveals that international migration is negligible among nurses, as well as in the total Brazilian population, which is 0.14%.

## Discussion

Regarding the sociodemographic profile, data on gender and age in this study are consistent with the results of Brazilian studies^(^ 12 ^-^ 14 ^)^. A study that characterized the sociodemographic profile of nurses qualified to enter the labor market in Minas Gerais, between 2005 and 2009, showed the predominance of female gender and age group of young adults, the most prevalent group being those less than 30, followed by those between 30 and 40 years^(^ 12 ^)^.

It should be noted that there are no current studies on the profile of Brazilian nursing in the national scenario; the last was conducted in 1982/1983. Research on the Nursing Profile in Brazil under development, in a collaboration between the Oswaldo Cruz Foundation, the Federal Nursing Council, the Brazilian Nursing Association and National Nurses' Federation, in order to know and analyze the profile of nursing categories^(^ 13 ^)^.

The findings of this study predominantly indicate three nurse migration flows: i) national migration driven by the training process with high levels of entrances and exits in states that concentrated numbers of courses and vacancies in undergraduate and *stricto sensu* graduate studies, especially in states with the highest increase in the supply of undergraduate vacancies during the year 2000. This movement is more evident among: states bordering one another, or between states of the same region of the country; ii) national migration motivated by job opportunity, especially in regions of economic growth of the country, and iii) international migration.

In the first flow, the data suggest entrances and exits in states that concentrated courses and vacancies in *stricto sensu* undergraduate and graduate courses, especially in the southeast. Studies in the country identified the concentrated supply of higher education institutions in regions with more development, which have a greater capacity in terms of qualified human resources and infrastructure. This concentration is evident in the distribution of courses supplied across regions of the country, but also within each region^(^ 9 ^-^ 14 ^)^. Thus, the southeast attracts students to nursing courses in other regions of the country, who then return, in most cases, to their countries of origin to obtain the title.

A study on the increased number of undergraduate nursing courses, given the movement of expansion of higher education in Brazil over the last two decades, showed that the expansion of nursing undergraduate courses was not accompanied by a study of the specific needs and demands of each region. This movement has reflected the logic of commodification of education, which considers the market needs and demands, and therefore does not contribute to meet the need for training of qualified professionals, aimed at resolving regional inequalities. The authors emphasize that it is necessary to increase the number of nurses in the country, ensuring that professionals are educated in recognized quality courses that can provide nurses with the profile and competence to intervene with proposals in the health care models^(^ 15 ^)^.

The second flow seems to be related to employment opportunities, as it is mainly directed to the country's regions with economic expansion and job supply. A few studies were identified on the labor market for nurses in Brazil. However, there is a trend of job instability and reduction of protected employment, increasing insecurity about the market, employment, income, employment and skills^(^ 5 ^)^.

The results of the study indicate that national migrants (both inter- and intra-state) are younger and most are single, individual factors contributing to the decision to migrate in search of opportunities. Those who migrate within their states have better job opportunities, since most engage in the profession and few are unemployed or remain outside the labor market. Those who migrate to other states have more difficulties, as shown by the higher proportion of unemployed and non-economically active.

In the third flow, the findings of the study indicate that the international flow mainly consists of returning migrants. It should be noted, when that compared to the other groups, international migrants are older, married in greater numbers, and engage least in the labor market. In this sense, the return to the country can be explained by familial factors.

Publications about international migration trends highlight the migration of nurses searching for employment opportunities and better salaries, among other factors^(^ 5 ^,^ 16 ^-^ 17 ^)^. A study conducted with migrant nurses in Ireland about their future intentions regarding migration, revealed that more than half of respondents considered migrating in the future, because the target country had failed to provide them with sufficient stability, especially in relation to citizenship and the ability for their family members to migrate with them. The factors outside the health system are reasons for respondents to migrate again^(^ 18 ^)^.

Another study, conducted in Chile, to investigate the cultural experiences of nurses who immigrated to the country, reported that in general, immigrant nurses identified as being satisfied with their professional achievements and with those related to their projects and lifestyles. In the process of establishing oneself as a nurse in the new country, the primary incentives consisted of the existence of employment opportunities, with some similarities in vocational education, and support on the part of patients. The difficulties were related to cultural and linguistic aspects, difficult relationship with colleagues, lack of health policies, and new responsibilities in care management^(^ 19 ^)^.

The migration of nurses can be defined by five attributes: the motivation and decisions of individuals, external facilitators and barriers, freedom of choice to migrate, the freedom to migrate as a human right, and dynamic movement. Migration background includes determining the political, social, economic, legal, historical and educational forces that comprise the forces of attraction and expulsion (*push and pull*). When considering its effects on the individual, the country of origin, the destination country, the health and nursing professional systems, the consequences of migration can be positive or negative (17-20). An integrative literature review found a need for more studies to answer questions about how and why nurses migrate^(^ 17 ^)^.

Migration flows occur due to influences of different socioeconomic conditions between countries, between regions within the same country, and within the same area. The movement of professionals is caused by the low economic development of countries and regions, the deficiencies in living conditions, and the lack of opportunities for professional development^(^ 8 ^)^. Migration flows occur to seek a labor market that better meets the demands of nurses, with better wages, geopolitical, cultural and linguistic conditions, equivalence in the level of training and education, and for individual factors^(^ 8 ^,^ 21 ^)^.

A systematic review on the migration of nurses working in educational institutions identified that the migration of this group differs from those of nurses working in direct care. According to the authors, the migration of nurse educators is a neglected issue that needs to be urgently investigated. The migration of nurses working in education is driven by better remuneration and infrastructure, which is influenced by institutional mobility programs^(^ 22 ^)^.

## Conclusion

The findings of this study enabled the development of a definition of the overall profile of the top-level workforce of nursing, with regard to migratory patterns. For this purpose, the sample data of the Demographic Census from 2010, with more than 30,000 cases identified as professional nurses, were consistent. In this sense, this study proved relevant to address, in a pioneering way, the possibility of using such data for analysis of migration in occupations that have high numbers of people. In the case of health care, the same exercise could be replicated, for example, with physicians and dentists.

It is suggested to deepen the discussion on the movement of nurses in Brazil and their migratory flows, especially in relation to motivating factors surrounding the decision to migrate. Qualitative research and focused surveys would allow the testing of the trends identified in this study, namely labor migration guided by the differentiated educational and work dynamics by country region. With specific regard to international migration, which was residual, research is suggested on its implications in establishing cooperation agreements, and compliance and recognition training. Finally, the possibility of data systematization for analysis of future projections of the labor force in nursing is indicated, using demographic techniques.
